# Three Therapeutic Strategies: Cinacalcet, Paricalcitol or Both in Secondary Hyperparathyroidism Treatment in Hemodialysed Patients During 1-Year Observational Study—A Comparison

**DOI:** 10.3389/fendo.2019.00040

**Published:** 2019-02-05

**Authors:** Jacek Zawierucha, Jolanta Malyszko, Jacek S. Malyszko, Tomasz Prystacki, Wojciech P. Marcinkowski, Teresa Dryl-Rydzynska

**Affiliations:** ^1^Fresenius Medical Care Polska S.A, Poznan, Poland; ^2^Department of Nephrology, Dialysis and Internal Medicine, Medical University of Warsaw, Warsaw, Poland; ^3^First Department of Nephrology and Transplantology With Dialysis Unit, Medical University of Bialystok, Bialystok, Poland

**Keywords:** cinacalcet, hemodialysis, outcome, paricalcitol, secondary hyperparathyroidism, vitamin D

## Abstract

**Introduction:** Secondary hyperparathyroidism (sHPT) is a common hormonal complication of chronic kidney disease. There are several therapeutic options for sHPT management aiming at calcium-phosphorus balance normalization and decrease of parathormone secretion.

**Objectives:** The aim of this retrospective, observational study was the outcome assessement of three most common therapeutic strategies of secondary hyperparathyroidism treatment with vitamin D receptor activator-paricalcitol, calcimimetic-cinacalcet or both agents administered together during in 12-months period.

**Methods:** One hundred and thirty-one haemodialysed patients with uncontrolled parathyroid hormone secretion have been treated with paricalcitol administered intravenously (group PAR−60 patients) or cinacalcet per os (group CIN−50 patients). The last group (group PAR+CIN−21 patients) received paricalcitol i.v. and oral cinacalcet administered simultaneously.

**Results:** In all groups, the iPTH level decreased significantly, however in group 1 treated with paricalcitol administered intravenously iPTH level decrease was greater than in group 2 treated with cinacalcet and in group 3 treated with paricalcitol and cinacalcet in parallel. The most substantial change of iPTH level was noticed after 3-months of observation. After this period the iPTH level was stabilized and maintained till the end of observation. Safety level of all strategies was comparable. No severe hypercalcemia or hypocalcemia was observed during the whole period of observation.

**Conclusions:** The results of observation show significant advantage of intravenous paricalcitol treatment. Complementing cinacalcet therapy with paricalcitol does not improve treatment outcomes. In case of unsatisfactory results after 3-months treatment, potential continuation should be considered carefully. Among three available therapeutic options, the treatment with paricalcitol i.v. should be considered in all haemodialysed patients with inadequate control of serum PTH level. The second option—with cinacalced administered orally should be considered in PD patients and when severe hypercalcemia occurs.

## Introduction

The number of patients with chronic kidney disease (CKD) requiring renal replacement therapy is growing every year. It makes secondary hyperparathyroidism (sHPT) a rising medical issue. SHPT is one of the most frequent hormonal complications connected with CKD, especially stage 4 and stage 5. Uncontrolled hyperparathyroidism contributes to the development and progression of mineral and bone disorder in chronic kidney disease (CKD-MBD)–serious complication leading to vascular and soft tissues calcification, abnormalities of bone turnover and mineralization, and decreased quality of life. There are several therapeutic strategies for sHPT management—from dietary restrictions which leads to limitation of phosphorus intake, through pharmacological intervention with phosphate binders, vitamin D or its analogs supplementation, calcimimetics administration to partial parathyroidectomy. According to the latest KDIGO (Kidney Disease: Improving Global Outcomes) guidelines limitation of “dietary phosphate intake in hyperphosphatemia treatment alone or in combination with other treatments” (2D level of evidence means that we suggest and the grade is very low) in CKD stages 3A−5D (dialysis) i.e., phosphate-binders are suggested (1). Moreover, restriction of calcium-based phosphate binders dose (2B level of evidence means that we suggest and the grade is moderate) is suggested as well. The KDIGO guidelines suggest “calcimimetics, calcitriol, or vitamin D analogs, or a combination of calcimimetics with calcitriol or vitamin D analogs” (with level of evidence 2B) to lower PTH in dialysis (1). In case of weak or lack of effects of pharmacological treatment partial or total parathyroidectomy should be considered. Data on the comparison of different SHPT treatment are very scarce and generally limited to studies on the effectiveness of different vitamin D analogs and vitamin D receptor analogs (VDRA).

The most popular intervention is supplementation with active vitamin D or the VDRA or calcimimetics administration. Administration of both agents (VDRA and calcimimetics) in parallel seems to be very promising due to opposite effects on serum calcium and different mechanism of action of drugs. However, in the literature the data on combination therapy are very limited. In the FARO-2 study this option was not analyzed due to low number of patients (2).

Taking all these data into consideration, we tried to analyze the real world data and compare efficacy of these therapeutic strategies, including combination treatment.

## Patients and Methods

This is an observational, retrospective study on 131 haemodialysed patients with inadequate control of PTH level treated with either intravenous paricalcitol (Paricalcitol Fresenius/Fresenius Medical Care Nephrologica)−60 patients aged 66 (*SD* = 11, Me = 66)–group PAR or cinacalcet (Mimpara/Amgen)−50 patients aged 60 (*SD* = 14, Me = 63)–group CIN. In the third group (PAR+CIN) of 21 patients aged 54 (*SD* = 13, Me = 51) treated with oral cinacalcet, intravenous paricalcitol has been added due to inability to reach target iPTH levels (more than 9 x higher than laboratory values limit), and then both agents were administered simultaneously.

The evaluation of the data, in particular, in the combination group was possible owing to introduction of therapeutic program for sHPT allowing to use either cinacalcet, paricalcitol or both agents. Every month of treatment iPTH, P, Ca, and ALP levels has been checked in all patients.

The treatment procedures were provided according to the therapeutic program approved by Polish Ministry of Health. Patients provided their informed consent for the therapeutic program. However, the authors also turned to the Ethical Committee of Regional Physicians Chamber in Poznan (Wielkopolska Izba Lekarska) and received their approval (Opinion no 89/2018).

Paricalcito doses were calculated according to Summary of Product Characteristics Paricalcitol Fresenius (3). Paricalcitol is a vitamin D3 analog. The chemical classification of paricalcitol is cholecalciferol. Chemical structure is (1R,3R)-5-[(2E)-2-[(1R,3aS,7aR)-1-[(E,2R,5S)-6-hydroxy-5,6-dimethylhept-3-en-2-yl]-7a-methyl-2,3,3a,5,6,7-hexahydro-1H-inden-4-ylidene]ethylidene]cyclohexane-1,3-diol.

The initial dose was established on the basis of serum iPTH concentration in pg/ml divided by 80. Subsequent doses were modified on the last iPTH serum concentration level checked monthly. The average dose of paricalcitol during whole observation period (12-months) was 6.76 mcg/dialysis session (Me = 5.00, Q1 = 3.84, Q4 = 35.00). Paricalcitol was administered intravenously to the bloodline during dialysis session. Cinacalcet is a naphthalene derivative and a calcium -sensing receptor agonist. Chemical structure is N-[(1R)-1-naphthalen-1-ylethyl]-3-[3-(trifluoromethyl)phenyl]propan-1-amine.The dosage of cinacalcet was based on Summary of Product Characteristics Mimpara Amgen (4) and the average dose during whole observational period was 0.6 mg/kg b.w. (*SD* = 0.3). Cinacalcet was taken by patient individually 2 times daily. Hyperphosphatemia was controlled by administration of calcium—based phosphate binders or non-calcium based phosphate binders as shown in Table 1. The dialysis was provided on Fresenius 5008S HD machines with FX Cordiax dialysers. The average dose of HD was 4 h three times a week.

**Table 1:** Clinical and demographic characteristics.

**Parameters**	**All (*n =* 131)**	**PAR (*n =* 60)**	**CIN (*n =* 50)**	**PAR+CIN (*n =* 21)**	***P*-value**
**AGE, YEARS**					
Mean (*SD*)	n/a	66 (11)	n/a	54 (13)	*p =* 0.01
Median	63	n/a	63	n/a	
Quartile 1	53	n/a	51.5	n/a	
Quartile 4	90	n/a	82	n/a	
**SEX**, ***n***					
Female	51	21	19	11	*p =* 0.3
Male	80	39	31	10	*p =* 0.3
**Use of phosphate binders, %**	42	45	40	40	*p =* 0.7
**Calcium containing only**	34	40	25	40	n/a
**Sevelamer only**	0	0	0	0	n/a
**Combination of calcium containing and sevelamer**	8	5	15	0	n/a
**Other binders**	0	0	0	0	n/a
**PARICALCITOL DOSE, mcg/HD SESSION**					
**Initial dose (week 0)**					
Mean	n/a	8.89	n/a	8.88	*p =* 0.06
Median		8.84		5.76	
Quartile 1		6.05		5.00	
Quartile 4		25.00		21.53	
**Week 12**					
Mean	n/a	7.19	n/a	10.64	*p =* 0.1
Median		5.00		7.69	
Quartile 1		5.00		4.46	
Quartile 4		30.00		35	
**Week 28**					
Mean (*SD*)	n/a	5.89	n/a	8.24	*p =* 0.2
Median		5.00		5.00	
Quartile 1		4.51		4.61	
Quartile 4		20.00		22.69	
**Week 52**					
Mean	n/a	4.74	n/a	4.94	*p =* 0.4
Median		4.23		4.61	
Quartile 1		3.08		3.07	
Quartile 4		27.69		18.84	
**CINACALCET DOSE, mg/kg B.W./DAY**					
**Initial dose (week 0)** Mean (*SD*)	n/a	n/a	0.6 (0.3)	0.6 (0.3)	*p =* 0.5
**Week 12** Mean (*SD*)	n/a	n/a	0.6 (0.3)	0.6 (0.3)	*p =* 0.5
**Week 28** Mean (*SD*)	n/a	n/a	0.6 (0.3)	0.6 (0.3)	*p =* 0.5
**Week 52** Mean (*SD*)	n/a	n/a	0.6 (0.3)	0.6 (0.3)	*p =* 0.5

The results are presented as percentage for categorical values, mean with one standard deviation in case of variables normally distributed. For non-normally distributed variables median and range were presented (tested by Lillefors test). The statistical significance was considered when *P*-value was < 0.05. For statistical significance assessment *T*-Test, One-Way Anova, Chi-Square, Wilcoxon, and Mann-Whitney tests were used accordingly.

## Results

**Figure 1:**
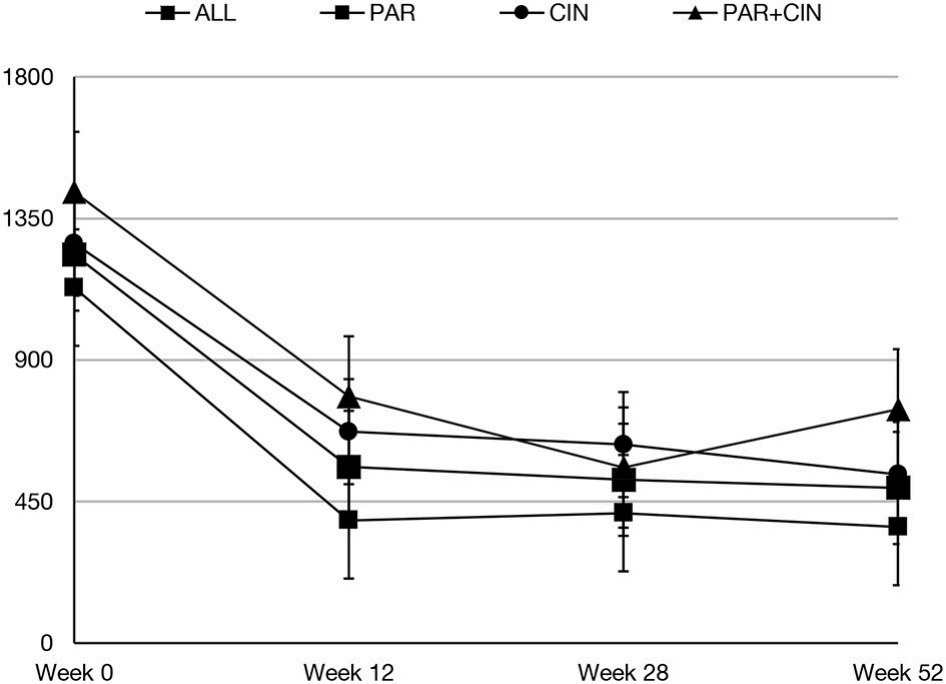
Serum iPTH concentration during observation period.

**Figure 2:**
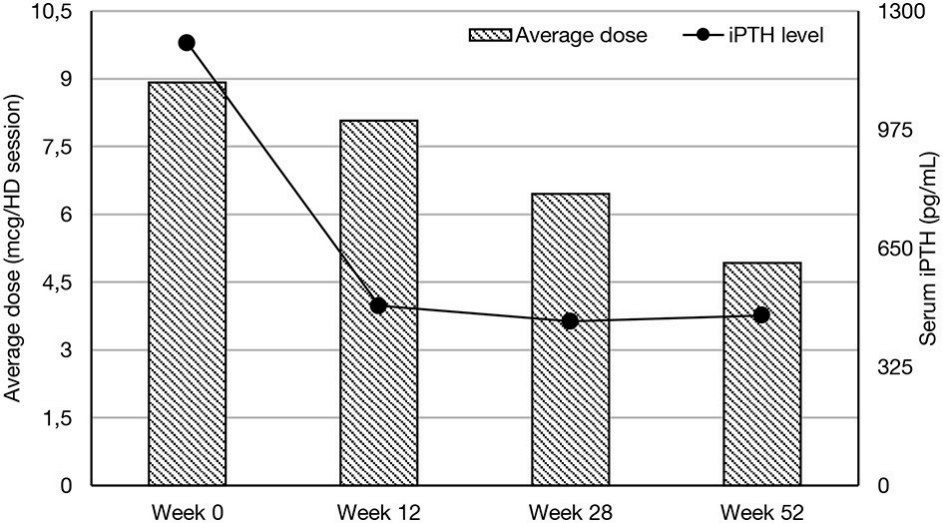
Average dose of paricalcitol during observation period.

In all groups iPTH serum level significantly decreased. The most substantial changes were observed in first period of observation (week 0–12). In the next period iPTH serum concentration stabilized and remained on the similar level till the end of observation, as shown on Figure 1. Changing the average paricalcitol dose based on the current iPTH level was easy and allowed to keep this parameter at the target level (see Figure 2). Comparison between all groups showed that the highest control of iPTH level is achievable with paricalcitol intravenous treatment. Statistically significant difference was observed between groups PAR and CIN in this parameter. Calcium and phosphate serum concentration changed in all groups during the period of observation, however no severe hypercalcemia or hypocalcemia was observed. Biochemical parameters during treatment are shown in Table 2.

**Table 2:** The effect of treatment on serum PTH, calcium, phosphate, and alkaline phosphatase.

		**iPTH (pg/mL)**		***P*-value**	**Ca (mg/dL)**		***P*-value**	**P (mg/dL)**			**ALP (U/L)**		
**Group**		**Week 0**	**Week 52**		**Week 0**	**Week 52**		**Week 0**	**Week 52**		**Week 0**	**Week 52**	
All	Average	1235	493	*p* < 0.0001	8.07	9.12	*p* < 0.0001	5.40	5,61	*p =* 0.13	199	120	*p* < 0.0001
	*SD*	–	–		–	–		1.49	1.60		–	–	
	Median	1072	339		8.56	9.20		–	–		125	84	
	Q1	863	228		7.84	8.47		–	–		87	63	
	Q4	2833	2490		16.00	11.52		–	–		1679	1211	
PAR (*SD*)	Average	1130	369	*p* < 0.0001	8.64	9.54	*p* < 0.0001	5.10	5.87	*p =* 0.002	151	88	*p* < 0.0001
	*SD*	–	–		0.90	0.75		1.36	1.47		–	–	
	Median	972	268		–	–		–	–		119	67	
	Q1	872	212		–	–		–	–		87	58	
	Q4	2472	1800		–	–		–	–		813	466	
CIN (*SD*)	Average	1271	536	*p* < 0.0001	7.13	8.49	*p =* 0.02	5.68	5.03	*p =* 0.02	627	380	*p =* 0.2
	*SD*	–	–		3.16	0.70		1.54	1.66		–	–	
	Median	1189	353		–	–		–	–		118	67	
	Q1	889	294		–	–		–	–		6	6	
	Q4	2500	2499		–	–		–	–		2833	2500	
PAR+CIN (*SD*)	Average	1434	743	*p =* 0.0005	8.65	9.40	*p =* 0.01	5.60	6.25	*p =* 0.08	367	107	*p =* 0.0001
	*SD*	–	–		1.02	0.96		1.57	1.37		–	–	
	Median	1443	720		–	–		–	–		198	72	
	Q1	795	330		–	–		–	–		102	63	
	Q4	1958	1688		–	–		–	–		1324	563	
*P*-value		*p =* 0.051	*p =* 0.0009	–	*p =* 0.006	*p* < 0.0001	–	*p =* 0.09	*p =* 0.002	–	*p =* 0.0007	*p =* 0.009	–

## Discussion

Secondary hyperparathyroidism and its consequences referred to as mineral and bone disorder in chronic kidney disease, despite new drugs and diagnostic methods development, remains an important factor of chronic kidney disease morbidity and mortality (5, 6).

Effectiveness and safety profile of sHPT treatment with calcimimetics as well as paricalcitol are widely described in literature (7–22).

However, there are no clear answers to key questions—whether sHPT management with active vitamin D or calcimimetics reduce mortality in HD population and which therapeutic approaches are most appropriate—vitamin D supplementation, and if so which of the available analogs is the most appropriate, the use of calcimimetics alone, or the administration of both preparations together. It is important to choose the form of administration—intravenously, during hemodialysis session or oral. Evidence for the reduction of mortality in hemodialysis patients receiving vitamin D supplementation origin mainly from observational studies. Several retrospective studies have identified the relationship between vitamin D administration in hemodialysis patients with chronic kidney disease in stage 5 and reduced mortality. A meta-analysis of the effects of active vitamin D on reducing mortality in patients with CKD in 2013 showed a significantly lower risk of death for any cause (23%) and a significantly lower risk of cardiovascular mortality (37% reduction) (23). Similarly, prospective FARO study showed statistically significant reductions in mortality from any cause and cardiovascular mortality in patients taking vitamin D analogs compared to untreated patients (24).

Therefore, when choosing the most beneficial treatment for a patient, the safety profile of the individual preparations and the mechanism of action must be taken into account.

Our study showed that in real life all employed strategies—oral cinacalcet, intravenous paricalcitol, and both agents administered simultaneously—are safe and efficient. The combination of cinacalcet and paricalcitol did not improve the efficacy, however cinacalcet might reduce vascular calcification as it was reported by others. There was no effect on serum calcium and phosphorus levels using the both agents. However, the combination of two drugs may be considered is some selected cases—e.g., hypocalcemia during treatment with cinacalcet, but in this particular case, the cost-effectiveness of such therapy should be carefully evaluated.

In several studies the comparison between paricalcitol and cinacalcet has been provided (25–29). In randomized IMPACT sHPT study cinacalcet was compared with paricalcitol in respect of iPTH reduction, serum calcium and phosphorus concentration changes, alkaline phosphatase activity, and FGF23 concentration. In 28 week observation, the iPTH level reduction was significantly higher in the group treated with paricalcitol. Additionally, the percentage of patients who reached the iPTH level in the range of 150–300 pg/ml was higher in this group (25–27). Incidence of adverse events during the 28 weeks of follow-up were comparable in both groups, however, paricalcitol use was associated with higher prevalence of hypercalcemia, whereas nausea and hypocalcemia were more frequent in the cinacalcet group (26). In addition, Sharma et al. (28, 29), on the basis of dosing and efficacy data from US patients involved to the IMPACT SHPT study, found that a strategy with paricalcitol administered intravenously was more cost effective than cinacalcet plus low-dose vitamin D in the management of PTH in patients with SHPT receiving hemodialysis.

Intravenous paricalcitol appears to be more cost-effective in hemodialysed patients with vascular access as well as has a better compliance. Oral cinacalcet would be more appropriate for patients with chronic kidney disease, including kidney transplant recipients treated conservatively and patients with CKD V treated with peritoneal dialysis. Adding cinacalcet to sHPT treatment protocol with iv paricalcitol can help to reduce hypercalcemic activity of paricalcitol however it was not showed in our study.

Data comparing head to head paricalcitol and cinacalcet are scarce. Chertow et al. (30) in open-label 16 weeks clinical trial, assess the effects of a treatment combined with low dose of active vitamin D derivatives and cinacalcet on mineral metabolism in patients on hemodialysis who had controlled PTH (iPTH 80–160 pg/ml) but remaining elevated Ca x P (>55 mg^2^/dl^2^) receiving paricalcitol >6 μg/week (or an equipotent dose of an alternative active vitamin D derivative). At the start of the trial, active vitamin D derivatives dose was decreased to average equivalent dose of paricalcitol 6 μg/week, and cinacalcet was titrated from 30 to 180 mg/day as a maximum possible dose. They concluded that treatment based on combination of low-dose active vitamin D derivatives and cinacalcet improved control of mineral metabolism. On the other hand, in this *post-hoc* analysis of ADVANCE study (31), coronary artery calcification progression was compared between 70 protocol-adherent subjects on cinacalcet and low doses of vitamin D (CPA) as specified in the study protocol and control group with 120 patients given vitamin D sterols. The study protocol stated specifically that the vitamin D dose was not to exceed the equivalent of 6 mcg of paricalcitol i.v. weekly among those receiving cinacalcet. Patients involved to the control group were treated with higher, varying doses of vitamin D sterols given intravenously during thrice-weekly hemodialysis sessions or orally every day. The authors found that the progression of CAC was weaker in the group treated with cinacalcet and small doses of vitamin D compared with the control group treated with higher doses of vitamin D sterols alone after 52 weeks. In the TARGET study on 444 hemodialysed patients with moderate to severe secondary parathyroidism (mean biointact PTH>160–430 pg/ml and approximately iPTH on the level 300–800 pg/ml or ng/l) the cinacalcet dose was titrated sequentially during an 8 weeks dose titration phase (30–180 mg/day) to get the bioPTH level below 160 pg/ml (iPTH 300 pg/ml or ng/l approximately) and the effectiveness was assessed over 8 weeks observation. At the second week of the trial, patients receiving vitamin S sterols get the reduced doses to the equivalent of 2 mcg of paricalcitol administered three times a week or 6 mcg administered in one dose weekly. Block et al. (32) concluded that the proportion of subjects with values of biPTH, of calcium x phosphorus product and of both biPTH and Ca x P within the target range during the analyzed period didn't show the difference between group received cinacalcet and vitamin D together and the group who was treated with cinacalcet alone. However, they did not assess the cost of this therapy. FARO 2 study showed that paricalcitol administered intravenously significantly increased the number of patients at the target for the combined endpoint composed of PTH, calcium and phosphate (*P* = 0.001), although the intravenous calcitriol and paricalcitol iv and cinacalcet combination groups weren't assessed due to the low patients number (2). In the retrospective study, Schumock et al. (33) compared rates of surgical intervention (parathyreoidectomy) in secondary hyperparathyroidism patients treated with paricalcitol or cinacalcet. They found that long-term treatment with paricalcitol was connected with lower number of parathyroidectomies in comparison with the patients treated with cinacalcet. It was obscure why patients in the cinacalcet group were more likely to experience parathyroidectomy in comparison to those who were treated with paricalcitol. The paricalcitol group consisted patients with more comorbidities, seemed to be more sicker and with a shorter period between the start of hemodialysis treatment and start of the index drug, while the cinacalcet group contained more females. Even when these discreptances and other ones were adjusted for in the final analysis, the risk of parathyroidectomy was markedly higher in the cinacalcet group. In our pilot study, we analyzed the results of 3-months paricalcitol treatment of 36 patients receiving hemodialysis with sHPT (serum iPTH >500 pg/ml), including 11 patients who additionally received cinacalcet. Analysis of the results shows a statistically significant lowering in iPTH and alkaline phosphatase in the whole group (34). In 2017 we published data (35) on 64 hemodialyzed patients with unsuited control of serum PTH levels treated for 6-months with intravenous paricalcitol, including 16 patients simultaneously receiving oral cinacalcet. In the first paper we collected all the available patients who entered the therapeutic programme with paricalcitol in FMC units. Till that time only treatment with cinacalcet was available. We had only 3-months complete data of a pilot study. Then we collected all the 6-months data from the growing HD population benefiting from the therapeutic programme. In both papers we had two study groups receiving either paricalcitol or paricalcitol with cinacalcet. In this study we were able to collect the biggest population of paricalcitol treated patients in Poland. We also were able to have 3 study group for comparisons i.e., paricalcitol, cinacalcet, and paricalcitol with cinacalcet. Moreover, we collected 1-year data.

Our study has several limitations, one is small sample size, nevertheless statistical analysis was feasible, contrary to FARO-2 study where the combination therapy sample was too small for analysis. We assessed only biochemical parameters, but not FGF23, we did not assess vascular calcifications. However, this is a real-world data. Intravenous paricalcitol, as well as combination of two drugs were not available for dialysis units until recently, when therapeutic program for sHPT and reimbursement by national Health Found were introduced. Now opportunity arises to assess the effect of this therapy, including combination of two drugs, clinical parameters and costs of the therapy as well. The results of EVOLVE have proven controversial (36–40). The unadjusted primary composite endpoint showed a non-significant reduction (HR: 0.93; P 1/4 0.112) with cinacalcet use (36–40). Both ADVANCE (31) and EVOLVE (36–40) trials evaluated the activity of cinacalcet on cardiovascular calcificationand the risk of cardiovascular incidents and mortality, respectively. Albeit the primary assay of both trials didn't find significant impact of cinacalcet, the benefit of one was insinuated in the subanalyses in which the potential issues of the trials were taken into account.

However, due to subsequent series of next publications of the EVOLVE study (36–40) KDIGO decided that calcimimetics, calcitriol or vitamin D analogs as well are capable first line strategy in G5D patients as agents active in PTH lowering. Authors didn't prioritized any option listed above. In principle, this recommendation was supposed to be maintained as it was in the previous set of KDIGO guidelines from 2009 (41). It is also worth to mention that there are alternative pathways of vitamin D (42–44) and lumisterol activation (45) that may affect the final outcomes.

## Conclusions

We report for the first time the long-term (1 year) efficacy data on combined therapy of sHPT in relation to either iv paricalcitol or oral cinacalcet. We are fully aware of limitation of our data, but they represent about 10% of Polish haemodialysed patients and therefore could be extrapolated to the larger population. The strength of the study is the same clinical protocol in all dialysis units, the same dialysers and same target goals on phosphate control, iPTH etc., possibility to access the data from the same system and to collect all available patients on combination therapy to make the comparison possible. Randomization of treatment allocation was not possible due to the retrospective analysis and this is clearly a limitation of this study. We are fully aware that allocation to either therapy would be the only way to convince readers and prevent from endless discussions about the results. However, there are no ongoing or registered studies on the comparison between cinacalcet and paricalcitol in the clinical.trials.gov. Thus, our not perfect, but real-life study show that both therapeutic strategies are effective in the treatment of sHPT, whereas simultaneous administration of the agents did not improve efficacy. It appears that low compliance of the patients in group treated with oral calcimimetics seems to be the main reason of weaker response in this group. In case of unsatisfactory results after 3-months treatment, the continuation of the therapy should be carefully considered. The treatment of sHPT with paricalcitol and cinacalcet is safe, however Ca concentration should be controlled during whole treatment period.

Cinacalcet is an effective agent in lowering iPTH level, but due to compliance issues should be considered mainly in the patients without vascular access (non-dialyzed CKD IV and V and patients receiving peritoneal dialysis) and in the patients with high Ca serum concentration to avoid hypercalcemia and all consequences connected with high calcium level.

Paricalcitol, due to intravenous administration should be considered in patients with vascular access (haemodialysed patients). Serum calcium level should be observed carefully and in case of severe hypercalcemia the treatment should be stopped, or dose of paricalcitol administered should be decreased.

## Author Contributions

JZ, JM, and JSM conceived the idea for the study and contributed to the design of the research. JZ performed the statistical analysis of the collected data. JZ, JM, and TD-R were involved in the preparation of the manuscript. JZ, JM, WM, and TP were involved in data collection. All the authors analyzed the data, edited and approved the final version of the manuscript.

### Conflict of Interest Statement

JZ, TP, WM, and TD-R are employees of Fresenius Medical Care. The manuscript was not submitted or published elsewhere. The remaining authors declare that the research was conducted in the absence of any commercial or financial relationships that could be construed as a potential conflict of interest.
